# On the Chemico-physiological Effects of Coffee

**Published:** 1854-01

**Authors:** Julius Lehmann


### *On the Chemico-physiological effects of Coffee.* By Julius Lehmann.

(Translated for the Examiner.)

Since the general introduction of coffee as a beverage, its mode of action upon the human organism has excited the attention and interest of many inquirers. Payen, several years ago, attempted to show its value as nourishment by the amount of nitrogen that it contained, starting, apparently, with the view that the nitrogen in a substance was the index of its nutritive power. As, however, it has been since ascertained that the determinaton of nitrogen for this purpose is only an index, when it is present in the form of the Protein compounds: and that as the Protein substance, the legumin, which is found in coffee only passes over in the smallest possible degree to the decoction, and that most of the nitrogen is due to the caffein, we are obliged to give up this view and seek another mode of explanation. Böcker, more lately, in order to explain its action, turned his attention to the urine passed after coffee had been used, and found that the secretion was thereby diminished. Lehmann, on the contrary, experimenting upon the action of caffein upon the organism, obtained an entirely opposite result, namely, an important increase of the secretion. With such contradictory views we are still left completely in the dark respecting a substance that exerts an immense influence upon the existence of many millions of beings.

The great consumption of coffee, as also its instinctive use by the poorer classes particularly, the most of whom are not in a condition to obtain, in proportion to their expenditure of strength, such an amount of plastic nourishment as is necessary for the preservation of health, led me to the view, that coffee, apparently, by retarding the metamorphosis of tissue in the body, renders what is an otherwise insufficient nourishment, sufficient.

I determined therefore, first to investigate the effects of the decoction of coffee upon the system generally, but more particularly upon its metamorphosis; afterward to examine its two characteristic constituents, caffein and empyreumatic oil, to discover to which of these its several actions are due.

In studying the metamorphosis in the system, nothing is more decisive than the examination of urine passed during a certain time; I have, therefore, confined myself principally to this point. As, however, important differences in the relative quantity of the constituents of urine are produced by trifling physiological conditions, for instance, by the quality and quantity of nourishment, physical exertion, mental emotions, &c., it will readily be perceived that a regular diet, and, as much as possible, a regular mode of life, is the first requisite in determining the effect of any substance upon the system. Several individuals should be subjected to the investigation, and care should be taken that it should not be renounced, if, after the first, second or third days, no reaction is perceived, or to conclude that it therefore exercises no effect. For I have frequently found that this reaction appeared first after several days. When it did take place I continued the investigation, until, by several uniform results, I could maintain with confidence its mode of operation. A great difficulty is removed if we succeed in obtaining such persons as are willing to conform to our necessarily strict demands. The only thing that could then stand in the way of the investigation would be their pathological conditions.

To examine now the effect of the decoction of coffee, and of its peculiar constituents upon the metamorphosis, I determine accurately the carefully collected quantity of urine passed in 24 hours, considering it sufficient to exhibit the three most important constituents, urea, chloride of sodium, and phosphoric acid.

The methods which I adopted are those given by Liebig, by which the urine has first attained its full importance, not only enabling the physician and chemist to execute a great series of experimonts in an extremely short space of time, and by most simple manipulations, but also possessing the important advantage of furnishing, especially by comparative analysis, the most accurate results, which was never possible by the old methods, in spite of the great loss of time. The methods for determining the chloride of sodium and urea contained in urine depend upon the following: If a weak solution of urea is mixed with a solution of the nitrate of oxide of mercury,* a white, flocky, curdy precipitate is instantly perceived, which soon becomes crystalline. This precipitate is a combination of the nitrate of urea with the oxide of mercury, $\overset{+}{Ur}\ NO_5{+}4\ HgO$. The nitrate of mercury is therefore precipitated by urea, but not the chloride of mercury. If a liquid contains, besides urea, also chloride of sodium, then upon the addition of nitrate of mercury, the white precipitate of urea will not occur until all the chloride of sodium present in the liquid has been decomposed by the nitrate of mercury into nitrate of soda and chloride of mercury. If more of the nitrate of mercury is now added to the liquid, then appears the precipitate of $\overset{+}{Ur}\ NO_5{+}4\ HgO$. As soon therefore, as from the further addition of nitrate of mercury, a cloudiness appears in the liquid, all the chloride of sodium is decomposed by it. Upon this is founded the determination of common salt in urine, which consists essentially of adding to the urine that is to be investigated a solution of nitrate of mercury of known strength, and observing how much thereof is used, before the permanent cloudiness arises. The quantity of the solution of nitrate of mercury thus used, gives the data for determining the amount of chloride of sodium contained in the liquid.

### *Determination of Urea.*

As above stated, a precipitate arises when a solution of nitrate of mercury is added to a solution of urea, containing 1 atom of nitrate of urea, $\overset{+}{Ur}\ NO_5$, to 4 atoms of oxide of mercury, HgO. If the amount of mercury added to the solution is exactly known, and if the limit is known when enough of it is added, so that the

*The equiv. of mercury is here taken, = 101.4 instead of 202.8, as adopted by Turner. The nitrate of the peroxide $Hg\,O_2\ 2\ NO_5 + HO$ (Mitscherlich,) is here intended.—(*Trans.*)

urea is all precipitated, then the amount of urea can be determined. A slight excess of nitrate of mercury added to the liquid to be investigated, can be detected by the yellow precipitate which it gives, when it is mixed on a watch glass with a solution of carbonate of soda. The first appearance of this yellow precipitate with carbonate of soda, is a certain indication that all the urea is completely precipitated. Since, however, the phosphates also give a white precipitate with oxide of mercury, their presence in the liquid would be a source of serious error, on which account they must always be removed before the determination of the chloride of sodium and urea. For this purpose 2 volumes of the urine must be measured out, and 1 volume of a mixture of a cold saturated solution of nitrate of baryta and aquæ baryta added thereto. Upon this the phosphate will be precipitated, the solution must now be filtered and the common salt and urea in the filtrate determined by the methods given above. For the determination of the phosphoric acid I made use of the method given by Liebig.

In the investigations, which I made upon two men, G. M. and H. S., my first object was to determine the normal average of *urine* passed in 24 hours, and of the amount of *urea*, *chloride of sodium*, and *phosphoric acid* contained in it, whilst on a diet, described further on, *without the use of coffee.* I continued the examination with this diet, until, for several days successively, the relative quantity of the three constituents remained about the same.

G. M., 32 years old, sound constitution, weighed 102 lbs. His diet in the last year corresponded with that of the poorer classes, which consists chiefly of bread, potatoes, much coffee, butter, frequently cheese, and seldom meat. His daily occupation, as well as his course of life, was on the whole very regular, so that he was well fitted for the present investigation. During the whole time he received daily the same quality and quantity of solid and liquid food. In the morning he received 4 oz. of white bread with butter, at noon 6 oz. of beefsteak with 4 oz. of boiled rice and as much bread; and in the evening 12 oz. of bread with butter. He drank during the day from 5 to 6 glasses of water, and in the evening 2 small glasses of beer. He continued this diet for several weeks, with the constant assurance that he oould live upon it throughout the year without weariness. The quantity of the solid products are represented in all the experiments by grammes.

*Results of the Investigation without the Use of Coffee.*

| Date. | Cub. Cent. Urine. | Reaction. | Peculiar Property. | Phosphoric acid. | Chloride of sodium. | Urea. |
|---|---|---|---|---|---|---|
| May | | | | | | |
| 19 | 1030 | acid. | clear, wine yellow. | 3.166 | 8.389 | 22.906 |
| 20 | 1130 | alkaline. | cloudy copious sediment. | 3.112 | 9.591 | 23.131 |
| 21 | 2020 | " | " " | 3.732 | 11.448 | 23.664 |
| 22 | 1808 | " | " " | 3.644 | 13.328 | 25.229 |
| 23 | 2010 | " | " " | 4.578 | 9.158 | 26.300 |
| 24 | 1695 | " | " " | 3.898 | 8.051 | 24.860 |
| 25 | 1284 | acid. | clear, wine yellow. | 3.842 | 9.714 | 24.598 |
| 26 | 1376 | " | " " | 4.423 | 9.420 | 25.282 |
| 27 | 1856 | " | " " | 5.796 | 12.341 | 25.096 |
| 28 | 1458 | " | " " | 4.238 | 9.429 | 27.360 |
| 29 | 1532 | " | " " | 4.156 | 8.892 | 26.250 |
| 30 | 1453 | " | " " | 3.998 | 9.245 | 26.975 |
| 31 | 1312 | " | " " | 4.098 | 10.001 | 27.996 |
| June | | | | | | |
| 1 | 1465 | " | " " | 4.210 | 9.251 | 27.584 |
| | 7220 | | | 20.700 | 46.818 | 136.150 |

H. S., 28 years old, of strong constitution, weighing 141 lbs., had been accustomed to tolerably strong food, at the same time in the daily use of coffee. His diet as well as his course of life were regulated during the examination like the other, with the difference only of receiving at noon 7½ oz. of meat. The results of the experiment upon his urine showed a corresponding increase of the three constituents.*

The disturbing effects which the entirely changed diet, particularly the withholding of coffee, produced upon the system, were exhibited more or less in both men, by a feeling of depression and debility, and were also shown in the cloudy, alkaline reacting urine, passed during the first days. G. M. exhibited the effects more strongly than H. S. After the system seemed to have accustomed itself to this mode of life, the urine again became entirely normal. The depressed feeling decreasing in both of them, but not entirely disappearing while on this diet. The average quantity of the three constituents I determined in these as well as in the following investigation, from the results which I ob-

*The tables referring to H. S.'s case are throughout omitted.—*Trans.*

tained after the system had returned to its normal condition and the important variation in the quantity of urine had ceased. It amounted to

G. M. per day 1444 CC urine, 4.140 $PO_5$ 9.363 Na CL 27.232 $\overset{+}{U}r$.

H. S. per day 1635 CC urine, 4.421 $PO_5$ 9·865 Na CL 31.298 $\overset{+}{U}r$.

To ascertain now the effects of the decoction of coffee upon the metamorphosis, these two persons continued the same diet, only with the difference, that instead of two glasses of water in the morning and two after dinner, they drank the decoction of coffee, each portion prepared from ¾ oz. of beans. From the first day of this diet, the feelings of depression disappeared. They became cheerful and well disposed for work. The urine remained entirely normal.

*Investigation of the Urine of G. M. after the the Use of Coffee.*

| Date. | Cub. Cent. Urine. | Reaction. | Peculiar Properties. | Phosphoric Acid. | Chl. of Sodium. | Urea. |
|---|---|---|---|---|---|---|
| June. | | | | | | |
| 3 | 2102 | acid. | wine yellow, clear. | 4.113 | 12.755 | 26.181 |
| 4 | 1480 | " | " " | 3.669 | 9.570 | 24.311 |
| 5 | 1620 | " | " " | 3.822 | 8.380 | 25.185 |
| 6 | 1500 | " | " " | 3.782 | 11.543 | 24.000 |
| 7 | 1846 | " | " " | 3.876 | 11.201 | 24.113 |
| 8 | 1520 | " | " " | 2.932 | 8.255 | 23.300 |
| 9 | 1340 | " | " " | 2.673 | 6.932 | 21.440 |
| 10 | 1620 | " | " " | 3.572 | 6.285 | 19.440 |
| 11 | 1560 | " | " " | 3.439 | 8.070 | 21.360 |
| 12 | 1520 | " | " " | 2.818 | 6.469 | 20.984 |
| 13 | 1322 | " | " " | 3.026 | 7.001 | 20.251 |
| | 7362 | | | 15.526 | 34.757 | 103.475 |

The same decrease was observed in H. S.'s case.

The average of urine and of its constituents passed in 24 hours amounted to,

In G. M. 1512 CC urine, 3.105 $PO_5$ 6.951 Na CL 20.695 $\overset{+}{U}r$.

In H. S. 2005 CC urine, 3.001 $PO_5$, 8.819 Na CL 21.888 $\overset{+}{U}r$.

From these results, it may be perceived that the decoction of coffee had but little influence upon the proportions of the three constituents, during the first days of the experiment. Its complete action was shown by G. M. on the 7th, and by H. S. on the 6th day of the investigation; the total amount of urine had increased, whilst a considerable diminution of the three constituents had taken place. It cannot be doubted, therefore that the main action of coffee is characterized by a retarding of metamorphosis.

To allow now the other action of coffee clearly to appear, I gave to each of these men a decoction of $1\frac{1}{2}$ oz. of coffee beans. Upon this appeared a greatly increased action of the heart, movement of the bowels, perspirations, anxiety and giddiness, which were followed by weakness, and sleep broken through unpleasant and confused dreams. The symptoms of its effects appeared as before in a stronger degree in G. M. than in H. S.

In the following experiments, my especial object was to ascertain to which of the two characteristic constituents, the caffein, or empyreumatic oil, the several actions of coffee must be ascribed, and especially which of these caused the retarding metamorphosis.

To ascertain first the effect of caffein, I again experimented upon G. M. I put him upon the same normal diet as before, until the quantity of the three constituents had attained their previous proportions, then gave him doses of caffein dissolved in water, in the morning and at noon, instead of the decoction of coffee.

*Investigation of the Urine of G. M. after the Use of Caffein.*

| Date. | Cub. Cent. Urine. | Reaction. | Peculiar Properties. | Phosphoric Acid. | Chl. of Sodium. | Urea. |
|---|---|---|---|---|---|---|
| June. | | | | | | |
| 20 | 1650 | Acid. | wine yellow, clear. | 4.013 | 8.690 | 26.413 |
| 21 | 1980 | " | " " | 3.925 | 11.387 | 25.843 |
| 22 | 2024 | " | " " | 3.742 | 9.376 | 25.264 |
| 23 | 2120 | " | " " | 3.854 | 10.967 | 24.199 |
| 24 | 1690 | " | " " | 3.821 | 8.557 | 24.231 |
| 25 | 1889 | " | " " | 3.643 | 9.101 | 23.901 |
| 26 | 2013 | " | " " | 3.756 | 9.561 | 24.023 |
| | 7712 | | | 15.074 | 38.186 | 96.354 |

The average per day was :

1928 CC. urine, 3,768,$PO_5$, 9.546 Na CL 24.088 $\overset{+}{U}$r.

Upon withdrawing coffee whilst in the use of the normal diet, G. M. experienced as before the same feeling of depression which completely left him, after the use of caffein.

The quantity of the three constituents, which after a few days of the normal diet had reached their previous amount, decreased again upon the use of the caffein, not, however, in such a degree as by the use of coffee. It may be concluded from this that there must be in coffee another constituent that retards the metamorphosis. No other effects of the daily use of 4 grains of caffein were perceived, except a somewhat increased action of the heart. I therefore, during the last day, gave to him 8 grains. This caused a quickened pulse, strong action of the heart, trembling and continued inclination to urinate, being able to pass but a small quantity. At the same time he showed a most excited state of mind, and afterwards, wandering thoughts, visions of a peculiarly intoxicated condition, which were followed by deep sleep. To establish satisfactorily the effect of caffein upon the metamorphosis, I gave also to another person under a similarly regulated diet 6 grains daily, and obtained the following results:

L. B., *with a diet without caffein* =1200 CC Urine.

7·790 Na CL 3.910 $PO_5$, 25·150 $\overset{+}{U}$r.

L. B. *with a diet with caffein* =1340 CC Urine,

6.890 Na CL 3·705 $PO_5$, 22·230 $\overset{+}{U}$r.

I delayed the investigation 8 days and began again.

A Diet *without caffein.*

1250 CC. Urine, 6.980 Na CL, 3.855 $PO_5$ 25.010 $\overset{+}{U}$r.

A Diet *with caffein.*

= 1276 CC. Urine, 6.790 Na CL, 3.690 $PO_5$ 20.800 $\overset{+}{U}$r.

From the above experiments, it may be asserted with tolerable confidence, that caffein does not *promote,* but rather *retards* the metamorphosis.—*Annal der Chem. und Pharm., Aug.* 1853.

(To be continued in our next number.)

